# Diversified Chaetoglobosins from the Marine-Derived Fungus *Emericellopsis* sp. SCSIO41202

**DOI:** 10.3390/molecules27061823

**Published:** 2022-03-11

**Authors:** Surun Shao, Xueni Wang, Jianglian She, Han Zhang, Xiaoyan Pang, Xiuping Lin, Xuefeng Zhou, Yonghong Liu, Yunqiu Li, Bin Yang

**Affiliations:** 1 CAS Key Laboratory of Tropical Marine Bio-Resources and Ecology/Guangdong Key Laboratory of Marine Materia Medica/Innovation Academy of South China Sea Ecology and Environmental Engineering, South China Sea Institute of Oceanology, Chinese Academy of Sciences, Guangzhou 510301, China; shaosurun@163.com (S.S.); wangxueni@scsio.ac.cn (X.W.); shejianglian20@mails.ucas.ac.cn (J.S.); xypang@scsio.ac.cn (X.P.); xiupinglin@scsio.ac.cn (X.L.); xfzhou@scsio.ac.cn (X.Z.); 2 Pharmacy School, Guilin Medical University, Guilin 541004, China; zhanghanmarc@163.com; 3 Southern Marine Science and Engineering Guangdong Laboratory (Guangzhou), Guangzhou 511458, China

**Keywords:** marine-derived fungus, *Emericellopsis* sp., chaetoglobosins, emeriglobosin

## Abstract

Two undescribed cytochalasins, emeriglobosins A (**1**) and B (**2**), together with nine previously reported analogues (**3**–**11**) and two known tetramic acid derivatives (**12**, **13**) were isolated from the solid culture of *Emericellopsis* sp. SCSIO41202. Their structures, including the absolute configurations of their stereogenic carbons, were fully elucidated based on spectroscopic analysis and the calculated ECD. Some of the isolated compounds were evaluated for their cytotoxicity and enzyme inhibitory activity against acetylcholinesterase (AChE) in vitro. Among them, **8** showed potent AChE inhibitory activity, with an IC_50_ value of 1.31 μM, and **5** showed significant cytotoxicity against PC-3 cells, with an IC_50_ value of 2.32 μM.

## 1. Introduction

Due to the high salinity, strong acidity, and high organic content of ecological conditions of mangroves, there is a very high diversity of microorganisms associated with mangrove sediments [1]. Until now, multiple different fungal species from mangrove sediment isolates have been cultured separately, including more classical fungal genera such as *Aspergillus* [2], *Penicillium* [3], *Lasiodiplodia* [4], and *Trichoderma* [5]. *Emericellopsis*, a new and rare fugal genus, was isolated from mangrove sediments for the first time. The genus *Emericellopsis* (Phycreales, Ascomycota) has more than 20 species, which are famous for their production of peptaibols with antibacterial and antifungal activities [6].

Alzheimer’s disease (AD) is a progressive and degenerative disorder of the brain associated with a reduction in the synaptic availability of acetylcholine (ACh). Therefore, increasing the synaptic levels of ACh in the brain by inhibiting the acetylcholinesterase (AChE) enzyme, which is primarily responsible for its hydrolysis and termination of action, is the most promising method for the symptomatic treatment of AD [7]. The most effective treatment of AD relies on three cholinesterase inhibitors (rivastigmine, donepezil, and galantamine) and memantine, which can affect the glutamatergic system but cannot cure AD, as they only lead to a temporary slowdown in the loss of cognitive function by decreasing cholinesterase activity [8]. The unsatisfactory effect of many current therapeutic drugs leads to an urgent need to find new drugs for AD. The diversity of different structures of chaetoglobosins could provide new lead compounds and templates for the future management of AD.

Chaetoglobosins are an important class of cytochalasin alkaloids isolated from various microorganisms, mainly from fungal species [9,10,11]. Structurally, all cytochalasins consist of 11-, 13-, or 14-membered carbocyclic (or oxygen-containing) rings connecting the C-8 and C-9 positions of a perhydroisoindol-1-one moiety bearing different substituents at C-3; in chaetoglobosins, the C-3 substituent is an (indol-3-yl)methyl group [12]. To date, more than 130 chaetoglobosins have been reported from the culture broths of some fungi, most belonging to the genus *Chaetomium* [13,14], and many of them have been reported to possess acute toxicity to mammals, cytotoxicity to human cancer cell lines, enzyme inhibitory activity, antibiotic activity, and phytotoxic activity, among others [15].

**Figure 1:**
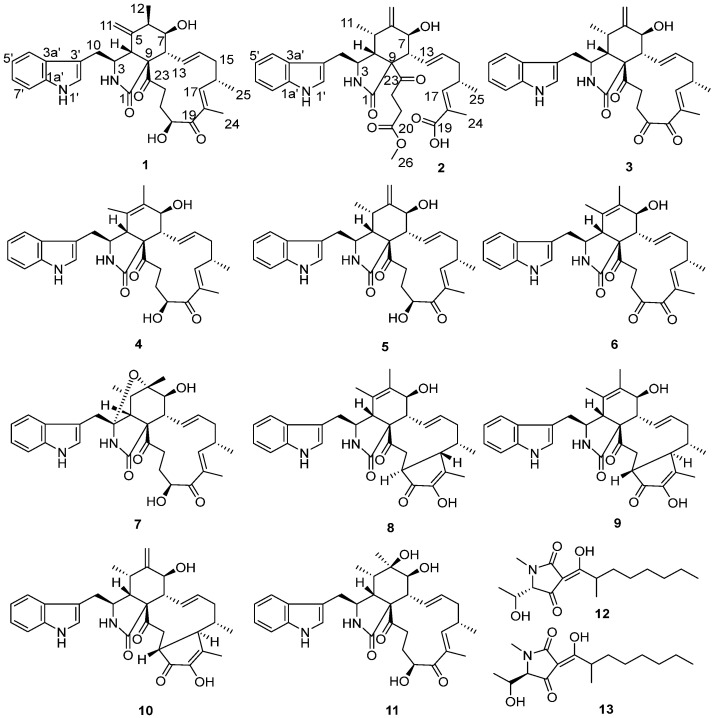
Chemical structures of compounds **1**–**13**.

During the course of our search for novel and bioactive compounds from fungi, the fungal strain *Emericellopsis* sp. SCSIO41202, isolated from a mangrove sediment sample from the South China Sea, furnished two new cytochalasin alkaloids, emeriglobosins A (**1**) and B (**2**), as well as nine previously described chaetoglobosins, viz., isochaetoglobosin D (**3**), chaetoglobosins E (**4**) [16,17], F_ex_ (**5**) [18], G (**6**) [16], W (**7**) [19], V(**8**) [19], and V_b_ (**9**) [20,21], cytoglobosin A (**10**) [22], armochaetoglobin S (**11**) [23], and two known tetramic acid derivatives, penicillenols A_1_ (**12**) [24,25] and A_2_ (**13**) [24,25] (Figure 1). Details of the isolation, structure elucidation, and biological activities of **4**–**13** are reported herein.

## 2. Results and Discussion

### 2.1. Structural Determination

**Figure 2:**
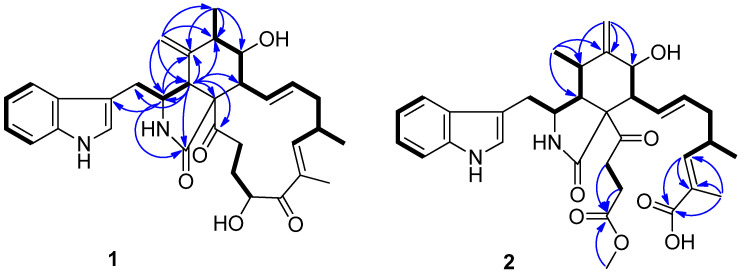
Selected HMBC and COSY correlations in **1** and **2**.

Compound **1** was isolated as a white amorphous powder. The HR-ESI-MS data suggested a molecular formula of C_32_H_38_N_2_O_5_ based on [M + Na]^+^ *m*/*z* 553.2662, indicating 15 degrees of unsaturation. The IR spectrum showed absorption bands at 3383 and 1682 cm^−1^, thereby implying the presence of amino and carbonyl groups. The aromatic proton signals at *δ*_H_ 7.52 (d, 7.9, H-4′), 7.04 (m, H-5′), 7.10 (m, H-6′), and 7.36 (d, 8.1, H-7′), along with an olefinic proton at *δ*_H_ 7.10 (m, H-2′), could be assigned to a 3-substituted indolyl group. The ^1^H and ^13^C NMR data of **1**, with the aid of DEPT and HSQC (Table 1 and Supplementary Materials) spectra, show the presence of three methyl groups, assigned to one singlet: H_3_-24 (*δ*_H_ 1.79, d, *J* = 1.1 Hz), and two doublets: H_3_-12 (*δ*_H_ 1.32, s) and H_3_-25 (*δ*_H_ 1.04, d, *J* = 6.7 Hz); four methylenes: H_2_-10 (*δ*_H_ 2.95, d, *J* = 5.4 Hz), H_2_-15 (*δ*_H_ 2.01, m; 2.45, m), H_2_-21 (*δ*_H_ 1.50, ddd, *J* = 14.4, 4.9, 2.7 Hz; 1.65, m), and H_2_-22 (*δ*_H_ 2.01, m; 2.45, m); seven methines: H-3 (*δ*_H_ 2.34–2.30, m), H-4 (*δ*_H_ 3.10, d, *J* = 4.6 Hz), H-6 (*δ*_H_ 2.34–2.30, m), H-7 (*δ*_H_ 3.30, dd, *J* = 11.3, 8.0 Hz), H-8 (*δ*_H_ 3.80, d, *J* = 4.8 Hz), H-16 (*δ*_H_ 2.79, m), and H-20 (*δ*_H_ 4.78, dd, *J* = 6.7, 4.7 Hz); and four olefines, attributed to H_2_-11 (*δ*_H_ 4.85, t, *J* = 1.4 Hz; 4.74, s), H-13 (*δ*_H_ 5.91, ddd, *J* = 15.1, 10.1, 1.9 Hz), H-14 (*δ*_H_ 5.18, ddd, *J* = 14.6, 11.1, 2.7 Hz), and H-17 (*δ*_H_ 6.26, m). Apart from the 23 aforementioned carbons, 9 non-protonated ones remained in the ^13^C-NMR spectrum, including 3 carbonyls (*δ*_C_ 175.0, 204.5, 207.3), 5 olefines (*δ*_C_ 108.6, 127.8, 135.1, 136.6, 148.3), and 1 oxygenated quaternary carbon (*δ*_C_ 64.7). These data suggest **1** belongs to a chaetoglobosin class. The NMR spectral data of **1** (Table 1) reveal a close similarity to those reported for chaetoglobosin Fex (**5**) [18]. The only difference between them was the position of the ethylidene group, which was established by the HMBC correlations (Figure 2) of H-11 with C-3, C-4, C-5, C-6, and C-12, and of Me-12 with C-5, C-6, and C-7, together with the proton spin systems from H-10/H-3/H-4 and Me-12/H-6/H-7/H-8/H-13/H-14/H-15/H-16(Me-25)/H-17 in the ^1^H−^1^H COSY spectrum. Therefore, the planar structure of **1** is shown in Figure 1.

**Figure 3:**
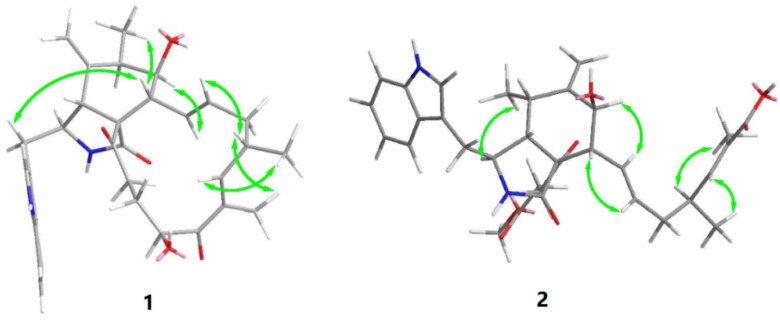
Key NOESY correlations in **1** and **2**.

Previous studies have suggested that the essential elements of the cytochalasin skeleton have the same stereochemistry, viz., the *cis*-stereochemistry across the 5/6 ring (*δ*_C_ 50.4, 64.7) junction, and the *trans*-stereochemistry of the macrocyclic ring [26,27]. The relative configuration of **1** was established by the analysis of the NOESY correlations, which were the same as those of the previously reported chaetoglobosins. In the NOESY spectrum, the relative configurations of C-6 and C-7 were assigned as *α*-oriented based on cross-peaks from H_3_-12/H-8/H-14 and H-7/H-13 (Figure 3). The *E*-geometry of Δ13 and Δ17 was deduced from a large coupling constant of *J*_13,14_ = 15.2 Hz and the NOESY correlation from H-16/18-Me, respectively. The absolute configurations of the stereogenic carbons in **1** were assumed to be 3*S*, 4*R*, 5*S*, 6*S*, 7*S*, 8*R*, 9*R*, 16*S*, and 20*S* based on its biogenic consideration. The calculated electronic circular dichroism (ECD) spectrum further confirmed the absolute configuration of **1** (Supplementary Materials).

Compound **2** is a white amorphous powder with the molecular formula C_33_H_40_N_2_O_7_, as deduced from an ion at *m*/*z* 577.2896 [M + H]^+^ in the HR-ESI-MS. The interpretation of the ^1^H and ^13^C NMR data (Table 1) of **2** revealed that the structure of **2** was quite similar to that of yamchaetoglobosin A [28]. The only difference in their ^13^C NMR spectra was that the keto carbonyl group in the latter was replaced by a carboxy carbonyl group (*δ*_C_ 170.4) at C-19 in **2**, which was supported by the HMBC correlations of H-17 with C-18 and C-19, and of Me-27 with C-17, C-18, and C-19 (Figure 2). The relative configuration of **2** was found to be the same as that of yamchaetoglobosin A by inspection of the NOESY correlations (Figure 3).

The structures of **3**–**13** were identified as isochaetoglobosin D (**3**); chaetoglobosins E (**4**) [16,17], F_ex_ (**5**) [18], G (**6**) [16], W (**7**) [19], V(**8**) [19], and V_b_ (**9**) [20,21]; cytoglobosin A (**10**) [22]; armochaetoglobin S (**11**) [23]; and penicillenols A_1_ (**12**) [24,25] and A_2_ (**13**) [24,25], by comparison of their HR-ESI-MS and NMR data with those reported in the literature.

### 2.2. Cytotoxic and Acetylcholinesterase (AChE) inhibitory Activities

**Figure 4:**
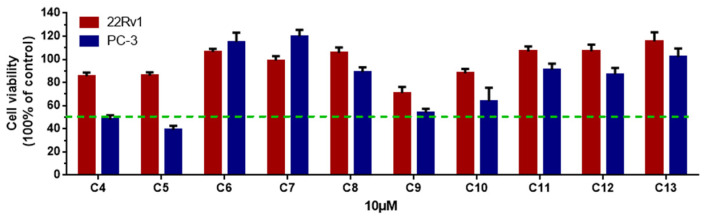
The cell viability of two prostatic carcinoma cell lines, PC-3 and 22Rv1, treated with **4**–**13** at 10 µM. All experiments were performed in triplicate.

**Figure 5:**
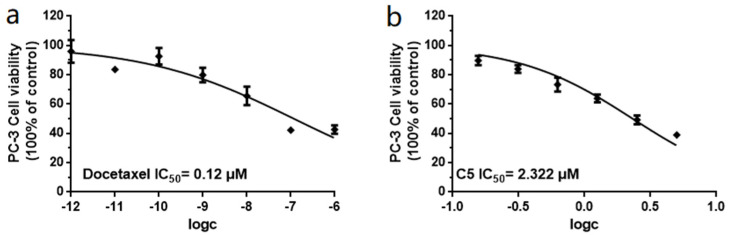
(**a**) IC_50_ values of docetaxel (positive control) against PC-3 cells. (**b**) IC_50_ values of **5** against PC-3 cells. All experiments were performed in triplicate.

Unfortunately, the amounts of **1**, **2**, and **3** were not sufficient to screen for prostate cancer cell growth inhibitory activity and enzyme inhibitory activity; therefore, we screened the remaining ten compounds (**4**–**13**) for their inhibitory activity against two prostatic carcinoma cell lines, PC-3 and 22Rv1 (Figure 4). None of the compounds showed activity against 22Rv1 cells; however, **5** showed the best inhibitory effect on PC-3 cells, with an IC_50_ value of 2.32 μM, and the positive control, docetaxel, had an IC_50_ value of 0.12 μM (Figure 5).

Additionally, the acetylcholinesterase (AChE) inhibitory activity of **4**–**13** was evaluated by in vitro experiments and in silico molecular docking analysis. The results indicate that **8** showed potent AChE inhibitory activity, with an IC_50_ value of 1.31 μM, whereas **4**–**6** and **9** exhibited moderate effects, with IC_50_ values of 4.15, 31.68, 8.71, and 23.66 μM, respectively, when compared with the positive control tacrine (IC_50_ = 0.02 μM).

**Figure 6:**
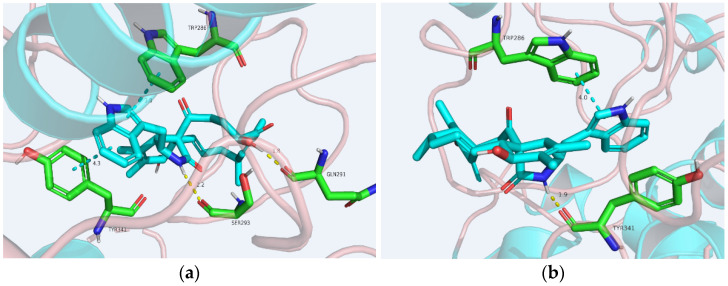
Proposed binding interactions of **4** (**a**) and **8** (**b**), with the active site residues of AChE (PDB ID: 4EY7). Yellow line: hydrogen bond; tiffany blue line: *π–π* stacking interaction.

In order to analyze the molecular interactions of the compounds, the active cytochalasins (**4**–**6**, **8**, **9**) were selected for docking into the AChE (PDB: 4EY7) active site. As a result, these molecules fit perfectly into the binding pocket with similar binding positions, with negative binding free energy values (S value) from −4.38 to −6.79. Compounds **4**–**6**, **8**, and **9** interacted with the AChE active site mainly through hydrogen bonds and *π–π* stacking interactions. The perhydroisoindol-1-one moiety’s *π–π* stacking with the residue Trp286 of protein 4EY7 interactions in most of the compounds was similar, but force sizes were different (Figure 6). OH-20 and NH-2 of **4** formed hydrogen bonds with Gln291 and Ser293, respectively. Meanwhile, **4** also formed *π–π* stacking with Tyr341. In particular, NH-2 of **8** formed hydrogen bonds with Tyr341. In addition, the perhydroisoindol-1-one moiety of **8** also interacted with Trp286 through *π–π* stacking. Nevertheless, all these interactions were beneficial for these compounds to anchor in the binding site of the enzyme. This docking is consistent with the in vitro AChE inhibitory activity performed in 96-well plates.

## 3. Materials and Methods

### 3.1. General Experimental Procedures

The NMR spectra were recorded on a Bruker AC 500 or AVANCE III HD 700 NMR spectrometer with TMS as an internal standard. HR-ESI-MS data were measured on a Bruker microTOF-QII mass spectrometer. Optical rotations were measured with an Anton Paar MCP500 polarimeter. UV spectra were obtained on a Shimadzu UV-2401PC spectrophotometer (Shimadzu Corporation, Kyoto, Japan). IR spectra were recorded on a Tensor 27 (Bruker Optics Gmbh, Ettlingen, Germany) with KBr pellets. CD spectra were measured with a Chirascan circular dichroism spectrometer (Applied Photophysics). YMC gel (ODS-A, 12 nm, S-50 µm) was used for column chromatography. The silica gel GF254 used for TLC was supplied by the Qingdao Marine Chemical Factory, Qingdao, China. Sephadex LH-20 gel (GE Healthcare, Stockholm, Sweden) was used. Semi-preparative HPLC was performed using an ODS column (YMC-pack ODS-A, YMC Co., Ltd., 10 × 250 mm, 5 µm, Kyoto, Japan). Spots were detected on TLC under UV light or by heating after spraying with 5% H_2_SO_4_ in EtOH (*v*/*v*). Artificial sea salt was a commercial product (Guangzhou Haili Aquarium Technology Company, Guangzhou, China). ODS (50 µm) was from Merk.

### 3.2. Fungal Material

*Emericellopsis* sp. SCSIO41202 was isolated from a mangrove sediment sample collected in Sanya (18°13′50.2″ N, 109°37′15.8″ E) in August 2017. The strain was identified as an *Emericellopsis* sp., based on a molecular biological protocol calling for DNA amplification and ITS region sequence comparison with the GenBank database, and shared a similarity of 99% with *Emericellopsis* sp. GYJ3(1) (accession N$\ddot{e}$ KM268654). The strain was deposited in the RNAM Center, South China Sea Institute of Oceanology, Chinese Academy of Sciences.

### 3.3. Fermentation and Extraction

The strain SCSIO41202 stored on PDA slants at 4 °C was cultured on PDA agar plates and incubated for 7 days at 28 °C in an incubator. Seed medium (infusion from 15 g of malt extract powder; sea salt, 2.5 g; distilled water, 1000 mL; pH = 7.4~7.8) in 150 mL Erlenmeyer flasks was inoculated with the fungus and incubated at 25 °C for 3 days on a rotating shaker (180 rpm). Autoclaved rice solid-substrate medium in 1000 mL flasks (rice, 200 g; sea salt, 6.6 g; distilled water, 220 mL) was inoculated with 10 mL seed solution. Flasks were incubated at 25 °C in a static position. After one month, cultures from 30 flasks were harvested for the isolation of substances. The obtained solid culture was crushed and extracted with twice the amount of acetone three times. The acetone extract was evaporated under reduced pressure to afford an aqueous solution, which was extracted with twice the amount of EtOAc to yield a crude gum (102 g).

### 3.4. Isolation and Purification

The EtOAc portion was subsequently separated by silica gel column chromatography using PET-CHCl_3_ gradient elution to obtain twenty-four fractions (Frs.1-24). Fr.13 was subjected to ODS gel column chromatography, using a gradient of MeOH (10%→100%) in H_2_O, to obtain 3 fractions (Frs.13-1~13-3). Frs.13-2 was purified by semi-preparative RP-HPLC (70% MeOH in H_2_O) at a flow rate of 3 mL/min to afford **12** (18.64 mg, t*_R_* = 39.2 min) and **13** (46.68 mg, t*_R_* = 43.8 min). Fr.15 was subjected to ODS gel column chromatography, using a gradient of MeOH (10%→100%) in H_2_O, to obtain 20 fractions (Frs.15-1~15-20). Frs.15-14 was purified by semi-preparative RP-HPLC (40% MeOH in H_2_O) at a flow rate of 3 mL/min to yield **3** (1.28 mg, t*_R_* = 30.2 min). Frs.15-15 was purified by semi-preparative RP-HPLC (40% MeCN in H_2_O) at a flow rate of 3 mL/min to offer **1** (1.30 mg, t*_R_* = 25.2 min), **2** (1.17 mg, t*_R_* = 26.5 min), and **7** (1.30 mg, t*_R_* = 23.3 min). Fr.16 was subjected to ODS gel column chromatography, using a gradient of MeOH (10%→100%) in H_2_O, to acquire 21 fractions (Frs.16-1~16-21). Frs.16-5 was separated on Sephadex LH-20 eluted with CH_2_Cl_2_–MeOH (*v*/*v*, 1:1) and then purified by semi-preparative RP-HPLC (53% MeOH in H_2_O) at a flow rate of 3 mL/min to provide **10** (9 mg, t*_R_* = 23 min), **11** (5.6 mg, t*_R_* = 34 min), and **5** (5 mg, t*_R_* = 39 min). Frs.16-10 was separated on Sephadex LH-20 eluted with CH_2_Cl_2_–MeOH (*v*/*v*, 1:1) and then purified by semi-preparative RP-HPLC (45% MeOH in H_2_O) at a flow rate of 3 mL/min to obtain **4** (2.6 mg, t*_R_* = 35 min). Frs.16-14 was separated on Sephadex LH-20 eluted with CH_2_Cl_2_–MeOH (*v*/*v*, 1:1) to afford two sub-fractons, Frs.16-14-2 and Frs.16-14-3. Additionally, Fr.16-14-2 was purified by semi-preparative RP-HPLC (45% MeOH in H_2_O) at a flow rate of 3 mL/min to offer **8** (1.7 mg, t*_R_* = 31 min). Fr.16-14-3 was purified by semi-preparative RP-HPLC (47% MeOH in H_2_O) at a flow rate of 3 mL/min to obtain **6** (5.1 mg, t*_R_* = 29 min), **7** (1.0 mg, t*_R_* = 33 min), and **9** (4.3 mg, t*_R_* = 43 min).

#### 3.4.1. Emeriglobosin A (**1**)

White amorphous powder; [*α*]${\;}_{25}^{D}$ −18 (c 0.01, MeOH); UV (MeOH) *λ*_max_ (log*ε*) 204 (6.33), 221 (6.37), 283 (6.48), 290 (6.49) nm; CD (0.30 mg/mL, MeOH) *λ*_max_ (log*ε*) 220 (−3.15), 269(0), 293 (−0.53) nm; IR (film) *ν*_max_ 3383, 2965, 2924, 1682, 1456, 1435, 1206, 1140 cm^−1^; ^1^H and ^13^C NMR data, Table 1; HRESIMS *m*/*z* 531.2848 [M + H]^+^ (calcd for C_32_H_38_N_2_O_5_, 531.2853), 553.2662 [M + Na]^+^ (calcd for C_32_H_38_N_2_NaO_5_, 553.2673).

#### 3.4.2. Emeriglobosin B (**2**)

White amorphous powder; [*α*]${\;}_{25}^{D}$ +37 (c 0.01, MeOH); UV (MeOH) *λ*_max_ (log*ε*) 204 (6.37), 221 (6.41), 282 (6.51), 290 (6.52), 371 (6.63) nm; CD (0.30 mg/mL, MeOH) *λ*_max_ (log*ε*) 209 (−0.76), 216 (−0.15), 225 (−0.25) nm; IR (film) *ν*_max_ 3375, 2963, 2920, 1682, 1541, 1506, 1456, 1435, 1339, 1206, 1138 cm^−1^; ^1^H and ^13^C NMR data, Table 1; HRESIMS *m*/*z* 577.2896 [M + H]^+^ (calcd for C_33_H_40_N_2_O_7_, 577.2908), 599.2723 [M + Na]^+^ (calcd for C_33_H_40_N_2_NaO_7_, 599.2728).

### 3.5. ECD Calculations

The relative configuration of **1** was subjected to random conformational searches using the Spartan’14 software with the MMFF method. The conformers with a Boltzmann population of over 5% (relative energy within 6 kcal/mol) were chosen for the ECD calculations using the Gaussian 09 software, and the stable conformers were initially optimized at the B3LYP/6-31+G (d, p) level in MeOH using the CPCM model. The overall theoretical calculation of the ECD was achieved in MeOH using time-dependent density functional theory at the B3LYP/6-31+G (d, p) level for the stable conformers of **1**. The ECD spectra of the different conformers were generated using SpecDis 1.6 (University of Würzburg) and Prism 5.0 (GraphPad Software Inc., San Diego, CA, USA) software, with a half-bandwidth of 0.3−0.4 eV, according to the Boltzmann-calculated contribution of each conformer after UV correction [29].

### 3.6. Bioactivity Assay

Compounds **4**–**13** were tested for their cytotoxicity against two prostatic carcinoma cell lines, PC-3 and 22Rv1, according to the reported CCK-8 (Dojindo) method. In brief, cells were seeded in a 96-well plate at the appropriate cell concentration and then incubated for 24 h at 37 °C in a 100% relative humidity, 5% CO incubator. Compounds to be tested were diluted to the appropriate concentration with culture medium, and the amount of each compound was adjusted to 25 μL before adding to the cells. Docetaxel (S1148, Selleck) was used as a positive control. The medium was aspirated, a fresh complete medium with 10% CCK-8 was added to incubate in a 37 °C incubator for 2-4 h, and the absorbance of the soultion at 450 nm was measured by an Envision 2104 multilabel reader (PerkinElmer). The inhibition rate was calculated by taking the absorbance at 650 nm as a reference [30].

The inhibitory effects of **4**–**13** on AChE were measured in vitro in 96-well plates as previously described [31]. Tacrine was used as a positive control, with an IC_50_ value of 0.02 μM.

### 3.7. Molecular Docking Analysis

The Schr$\ddot{o}$dinger 2017-1 suite was employed to perform docking analyses [32]. Several three-dimensional crystal structures of AChE (PDB code: 1H22, 1H23, 4EY7, 5EI5) were retrieved from a protein data bank and constructed following the Protein Prepare Wizard workflow in the Maestro package. The binding site was determined by the Grid Generation procedure. The prepared ligand was then flexibly docked onto the receptor using Glide (XP mode) with default parameters.

## 4. Conclusions

In brief, chemical investigation of a culture of the mangrave sediment-derived fungus *Emericellopsis* sp. SCSIO41202 resulted in the isolation of two unreported cytochalasins, emeriglobosins A (**1**) and B (**2**), together with nine known analogues (**3**–**11**) and two known tetramic acid derivatives (**12, 13**). Compound **5** exhibited significant inhibitory activity against PC-3 cells, with an IC_50_ value of 2.32 μM. Compound **8** showed potent AChE inhibitory activity, with an IC_50_ value of 1.31 μM, whereas **4**–**6** and **9** exhibited moderate effects, with IC_50_ values of 4.15, 31.68, 8.71, and 23.66 μM, respectively. Collectively, this work expands the family of cytochalasins and provides more drug lead compounds with potential medicinal value.

## Figures and Tables

**Table 1:** ^1^H (700 MHz) and ^13^C (175 MHz) NMR spectroscopic data of **1** and **2** in CD_3_OD.

	1		2	
No.	*δ*c, Type	*δ*_H_ (*J* in Hz)	*δ*c, Type	*δ*_H_ (*J* in Hz)
1	175.0, C		174.9, C	
3	50.9, CH	2.34–2.30, m	53.0, CH	3.50, q (5.3)
4	50.4, CH	3.10, d (4.6)	46.6, CH	2.77, m
5	148.3, C		31.5, CH	2.95, dd (14.5,61)
6	45.7, CH	2.34–2.30, m	150.0, C	
7	72.4, CH	3.30, dd (11.3, 8.0)	71.1, CH	3.91, m
8	58.1, CH	3.80, d (4.8)	48.3, CH	2.88, dt (19.5,7.3)
9	64.7, C		63.2, C	
10	30.2, CH_2_	2.95, d (5.4)	31.7, CH_2_	2.88, dt (19.5,7.3) 2.81, d (9.3)
11	114.6, CH_2_	4.85, t (1.4) 4.74, s	12.8, CH_3_	0.89, d (6.8)
12	21.6, CH_3_	1.32, s	112.3, CH_2_	5.26, s 5.05, s
13	127.9, CH	5.91, ddd (15.1, 10.1, 1.9)	128.7, CH	5.83, dd (15.2,9.5)
14	134.2, CH	5.18, ddd (14.6, 11.1, 2.7)	132.6, CH	5.35, dt (14.8,7.0)
15	40.5, CH_2_	2.45, m 2.01, m	39.3, CH_2_	2.06, d (7.3)
16	33.2, CH	2.79, m	33.3, CH	2.57, dq (13.6,6.8)
17	148.7, CH	6.26, m	147.7, CH	6.55, d (10.0)
18	135.1, C		126.3, C	
19	204.5, C		170.4, C	
20	70.6, CH	4.78, dd (6.7, 4.7)	173.4, C	
21	30.6, CH_2_	1.65, m 1.50, ddd (14.4,4.9,2.7)	26.9, CH_2_	2.24, tt (12.8,6.2) 2.17, ddd (25.1,11.4,5.8)
22	36.4, CH_2_	2.45, m 2.01, m	34.9, CH_2_	2.87, d (9.3)
23	207.3, C		207.8, C	
24	10.9, CH_3_	1.79, d (1.1)	11.2, CH_3_	1.80, m
25	18.7, CH_3_	1.04, d (6.7)	18.4, CH_3_	0.98, d (6.7)
26			50.7, CH_3_	3.66, s
1’a	136.6, C		136.6, C	
2’	124.0, CH	7.10, m	124.1, CH	7.14, s
3’	108.6, C		109.3, C	
3’a	127.8, C		127.7, C	
4’	117.7, CH	7.52, d (7.9)	118.0, CH	7.55, d (7.9)
5’	118.6, CH	7.04, m	118.6, CH	7.04, t (7.2)
6’	121.1, CH	7.10, m	121.0, CH	7.10, t (7.3)
7’	111.1, CH	7.36, d (8.1)	111.0, CH	7.33, d (8.1)

## Data Availability

All data and figures in this study are openly available.

## Supplementary Materials

The following supplementary materials can be downloaded at: https://www.mdpi.com/article/10.3390/molecules27061823/s1, Figures S1–S9: ^1^H, ^13^C-NMR, HSQC, HMBC, COSY, NOESY, UV, IR and HRESIMS spectra of compound **1**; Figures S10–S17: ^1^H, ^13^C-NMR, HSQC, HMBC, NOESY, UV, IR and HRESIMS spectra of compound **2**; Figure S18: CD of compounds **1** and **2**; Figure S19: Comparison between experimental ECD spectra and calculated ECD spectra of **1**. Figures S20–S21: Molecular docking of **5**, **6**, and **9** with 4EY7.

## Sample Availability

Samples of the compounds **1**–**13** are available from the authors.
