# Supplementary material for: Diversified Chaetoglobosins from the Marine-Derived Fungus Emericellopsis sp. SCSIO41202
Source: Molecules. 2022 Mar 11;27(6):1823. doi: 10.3390/molecules27061823 (PMC8948984; doi:10.3390/molecules27061823)
Supplement: Supplementary file 1 [file molecules-27-01823-s001.zip › molecules-1576759-supplementary.pdf]

Diversified Chaetoglobosins from the marine-derived fungus *Emericellopsis* sp. SCSIO41202

Surun Shao[2], Xueni Wang[1], Jianglian She [1], Han Zhang[2], Xiaoyan Pang[1], Xiuping Lin[1,3], Xuefeng Zhou[1,3], Yonghong Liu[1,3]*, Yunqiu Li[2]* and Bin Yang[1,3]*

[1] CAS Key Laboratory of Tropical Marine Bio-resources and Ecology/Guangdong Key Laboratory of Marine Materia Medica/Innovation Academy of South China Sea Ecology and Environmental Engineering, South China Sea Institute of Oceanology, Chinese Academy of Sciences, Guangzhou 510301, P. R. China; wangxueni@scsio.ac.cn (X.-N.W.); shejianglian20@mails.ucas.ac.cn (J.L. S.); xypang@scsio.ac.cn (X.-Y.P.); xiupinglin@scsio.ac.cn (X.-P.L.); xfzhou@scsio.ac.cn (X.-F.Z.)

[2] Pharmacy School of Guilin Medical University, Guilin 541004, P. R. China; shaosurun@163.com (S.-R.S.); zhanghanmarc@163.com (H. Z.)

[3] Southern Marine Science and Engineering Guangdong Laboratory (Guangzhou), Guangzhou 511458, China

* Correspondence: yangbin@scsio.ac.cn (B.Y.); Tel.: +86-20-89023174; leeyq88@126.com (Y.-Q. L.); yonghongliu@scsio.ac.cn (Y.-H.L.)

Contents of Supporting Information

Figure S1. ^{1}H NMR of compound **1** (in CD_3OD)..3

Figure S2. ^{13}C NMR of compound **1** (in CD_3OD)..3

Figure S3. HSQC of compound **1** (in CD_3OD)..4

Figure S4. HMBC of compound **1** (in CD_3OD) 4

Figure S5. ^{1}H-^{1}H COSY of compound **1** (in CD_3OD)..5

Figure S6. NOESY of compound **1** (in CD_3OD)...5

Figure S7. HRESIMS of compound **1** 6

Figure S8. UV of compound **1**..7

Figure S9. IR of compound **1**...8

Figure S10. ^{1}H NMR of compound **2** (in CD_3OD)...8

Figure S11. ^{13}C NMR of compound **2** (in CD_3OD)..9

Figure S12. HSQC of compound **2** (in CD_3OD)..9

Figure S13. HMBC of compound **2** (in CD_3OD) 10

Figure S14. NOESY of compound **2** (in CD_3OD)..10

Figure S15. HRESIMS of compound **2** 11

Figure S16. UV of compound **2**...12

Figure S17. IR of compound **2**..13

Figure S18. CD of compounds **1** and **2**…………………………………….…………...…13

Figure S19. Molecular docking of chaetoglobosin Fex………………………….……..……14

Figure S20. Molecular docking of chaetoglobosin G……………………………….……..……14

Figure S21. Molecular docking of chaetoglobosin Vb……………………….……....……14

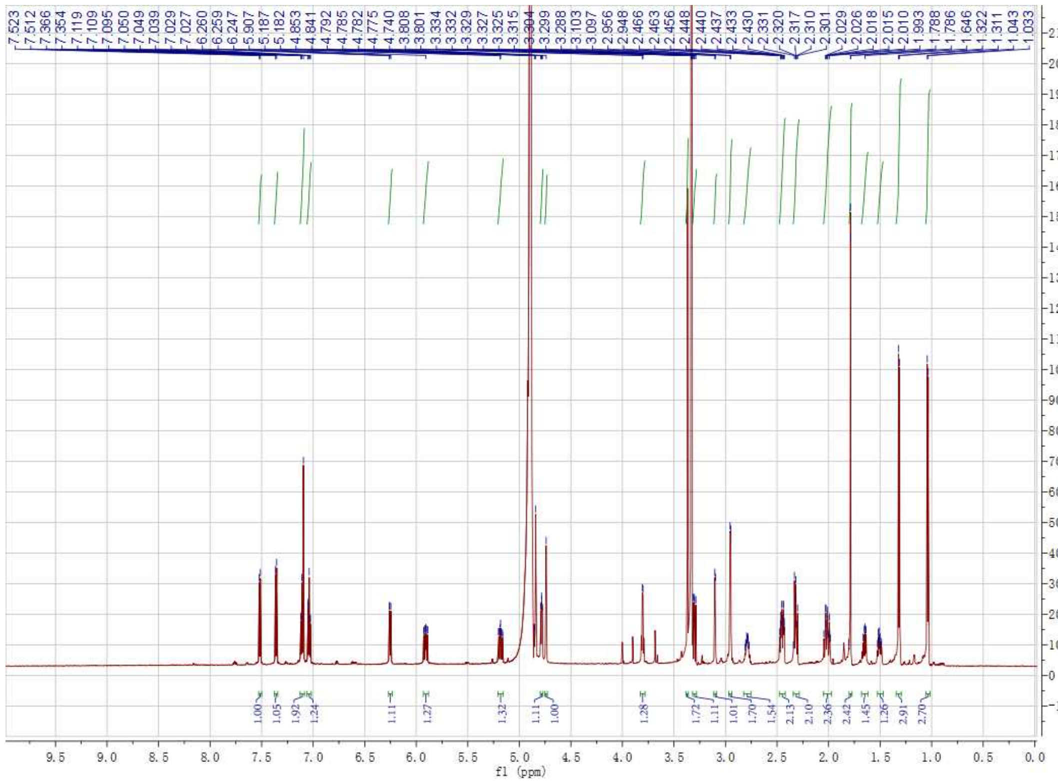

Figure S1. ^{1}H NMR of compound **1** (in CD_3OD)

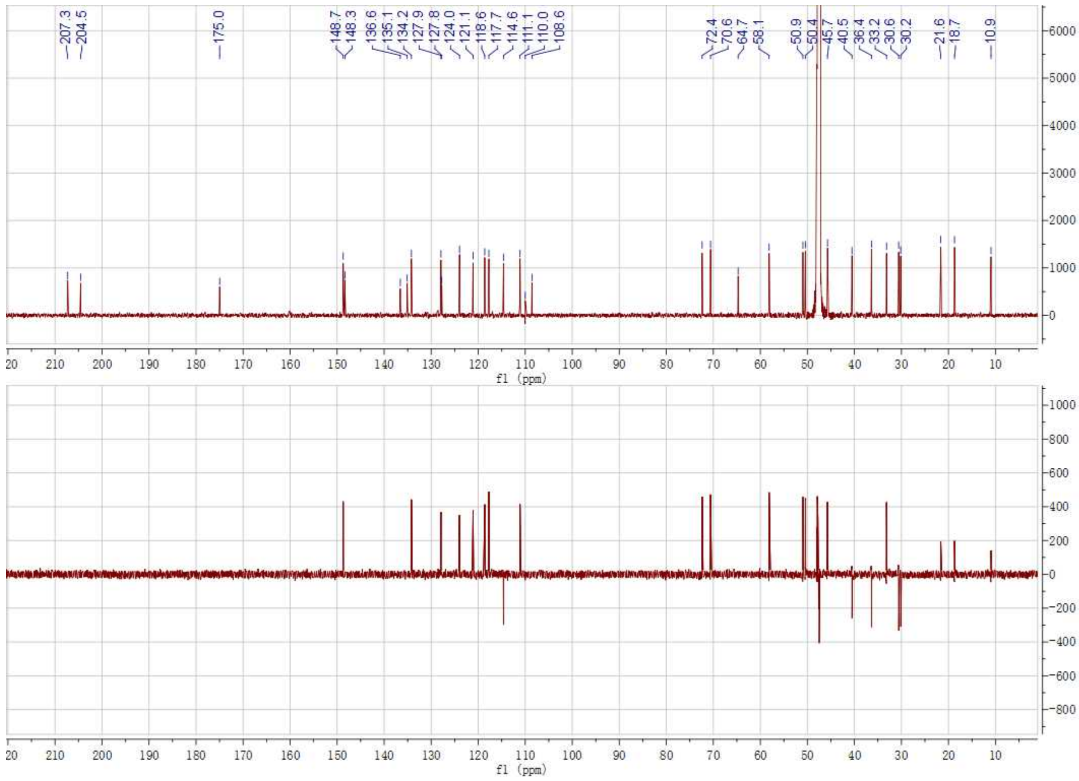

Figure S2. ^{13}C NMR of compound **1** (in CD_3OD)

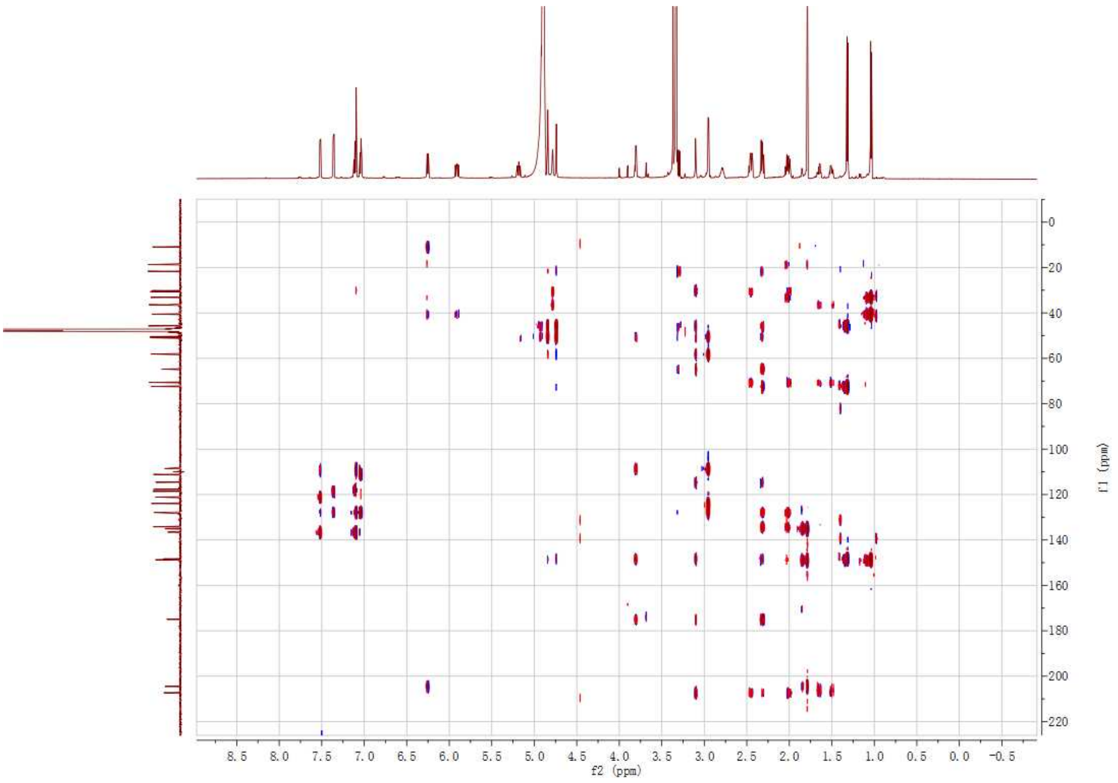

Figure S3. HSQC of compound **1** (in CD_3OD)

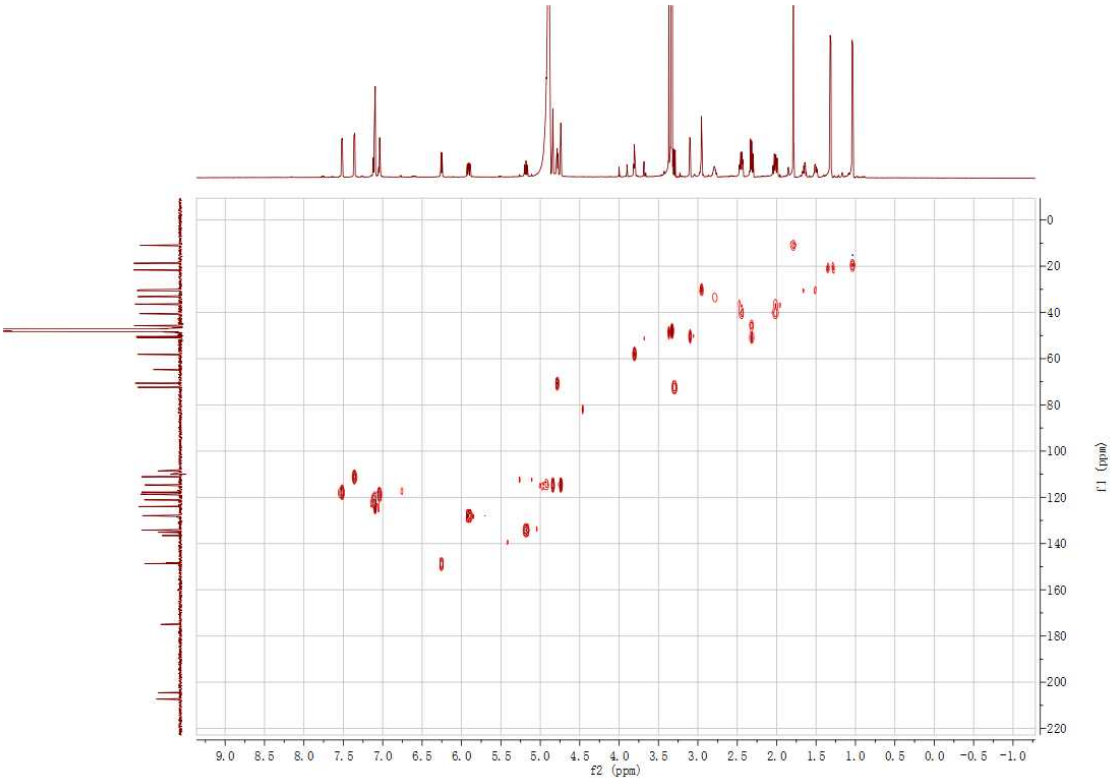

Figure S4. HMBC of compound **1** (in CD_3OD)

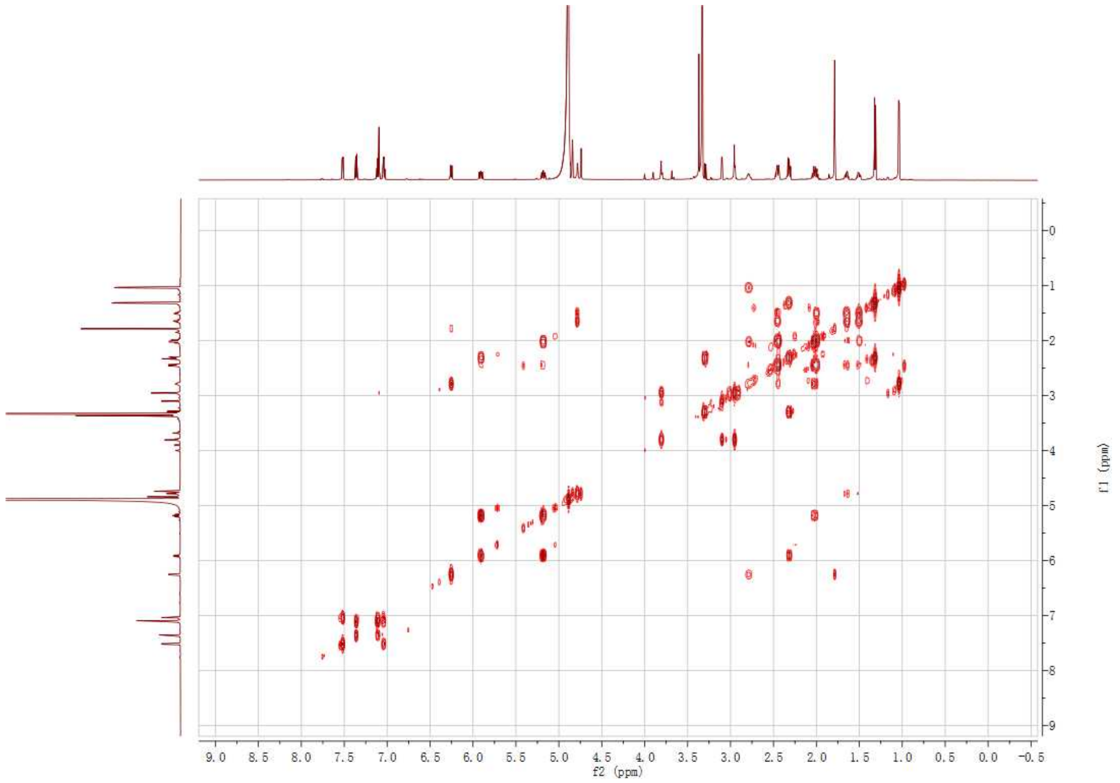

Figure S5. ^{1}H-^{1}H COSY of compound **1** (in CD_3OD)

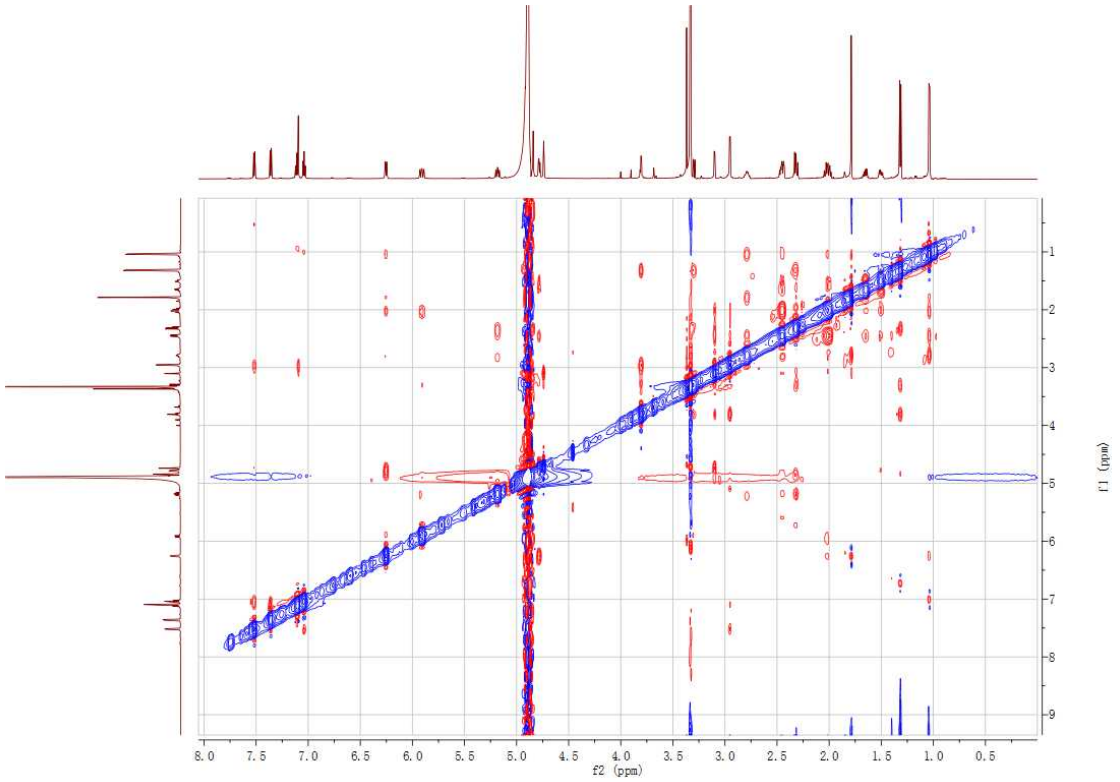

Figure S6. NOESY of compound **1** (in CD_3OD)

Mass Spectrum SmartFormula Report

Analysis Info

Analysis Name	D:\Data\MS\data\202107\shaosurun_LTH1-15-2_pos_51_01_11333.d	Acquisition Date	8/2/2021 3:01:06 PM
Method	LC_Direct Infusion_pos_70-500mz.m	Operator	SCSIO
Sample Name	shaosurun_LTH1-15-2_pos	Instrument	maXis 255552.00029
Comment			

Acquisition Parameter

Source Type	ESI	Ion Polarity	Positive	Set Nebulizer	0.4 Bar
Focus	Active	Set Capillary	4500 V	Set Dry Heater	180 °C
Scan Begin	70 m/z	Set End Plate Offset	-500 V	Set Dry Gas	4.0 l/min
Scan End	1500 m/z	Set Charging Voltage	0 V	Set Divert Valve	Waste
		Set Corona	0 nA	Set APCI Heater	0 °C

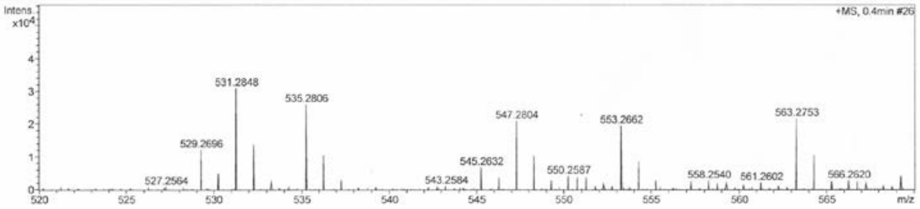

Meas. m/z	#	Ion Formula	Score	m/z	err [ppm]	err [mDa]	mSigma	rdb	e⁻ Conf	N-Rule
531.2848	1	C32H39N2O5	100.00	531.2853	1.1	0.6	43.0	14.5	even	ok
553.2662	1	C32H38N2NaO5	100.00	553.2673	2.0	1.1	50.9	14.5	even	ok

shaosurun_LTH1-15-2_pos_51_01_11333.d

Bruker Compass DataAnalysis 4.1 printed: 8/2/2021 3:18:39 PM by: SCSIO Page 1 of 1

Figure S7. HRESIMS of compound **1**

光谱峰值检测报告

2021/07/02 16:43:10

数据集: LTH1-15-2 - RawData

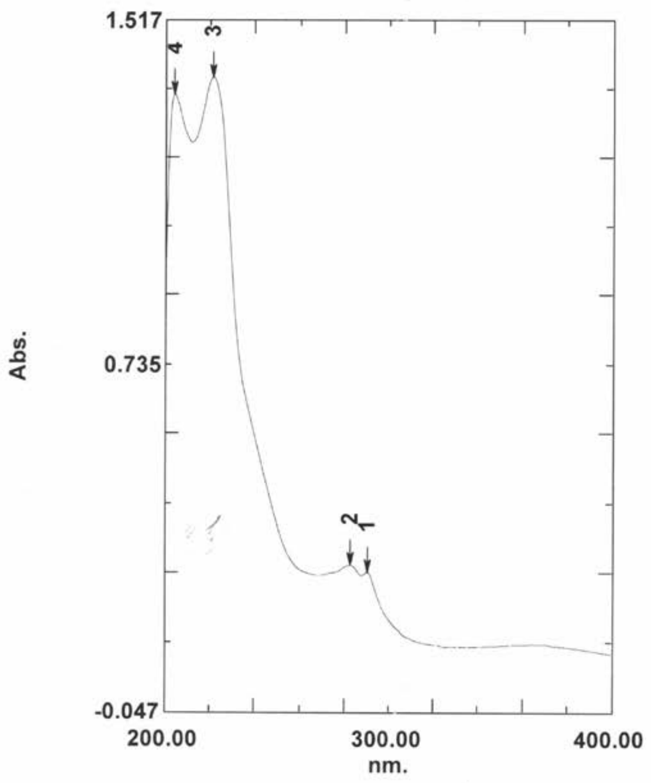

[测定属性]
波长范围(nm): 200.00 到 400.00
扫描速度: 中速
采样间隔: 0.2
自动采样间隔: 启用
扫描模式: 单个

[仪器属性]
仪器类型: UV-2600 系列
测定方式: 吸收值
狭缝宽: 2.0
积分时间: 0.1 秒.
光源转换波长: 323.0 nm
检测器单元: 直接
S/R 转换: 标准
阶梯校正: OFF

[附件属性]
附件: 无

[数据处理参数]
阈值: 0.0010000
点: 4
内插: 停用
平均: 停用

[样品准备属性]
重量:
体积:
稀释:
光程长:
附加信息:

No.	P/V	波长(nm)	Abs.	描述
1	↑	290.20	0.267	
2	↑	282.60	0.282	
3	↑	221.20	1.386	
4	↑	204.00	1.346	

Figure S8. UV of compound **1**

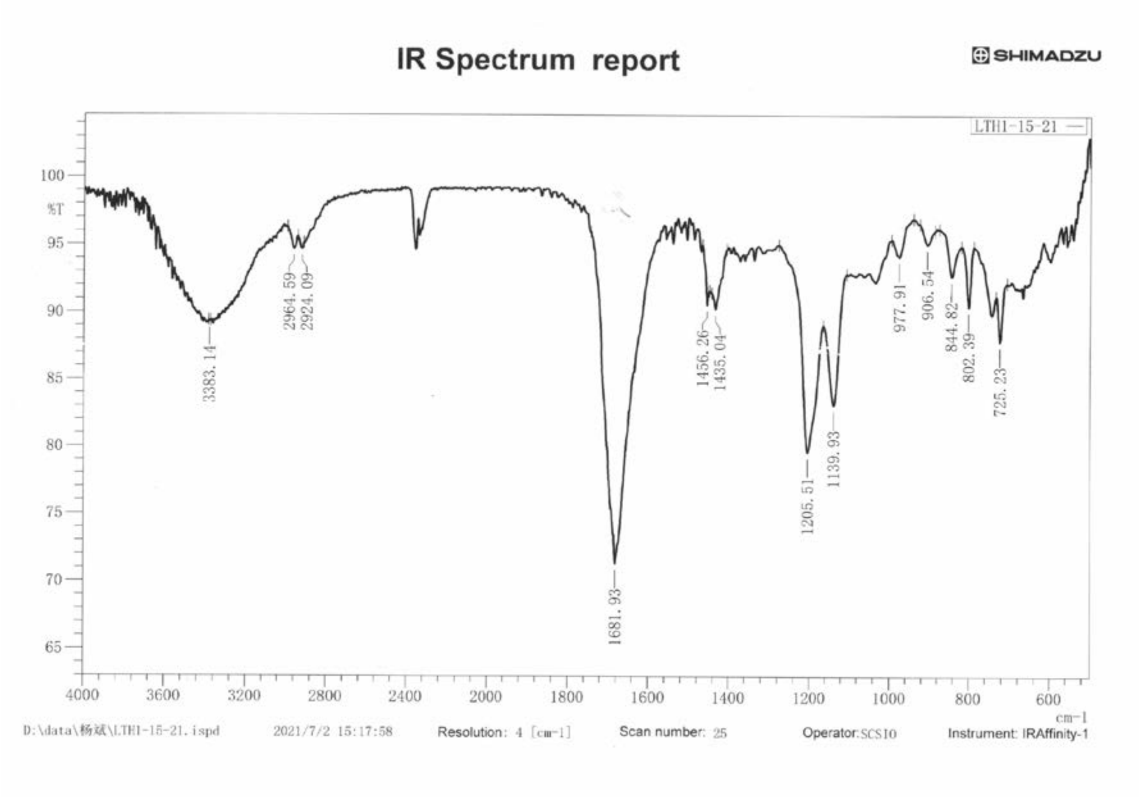

Figure S9. IR of compound **1**

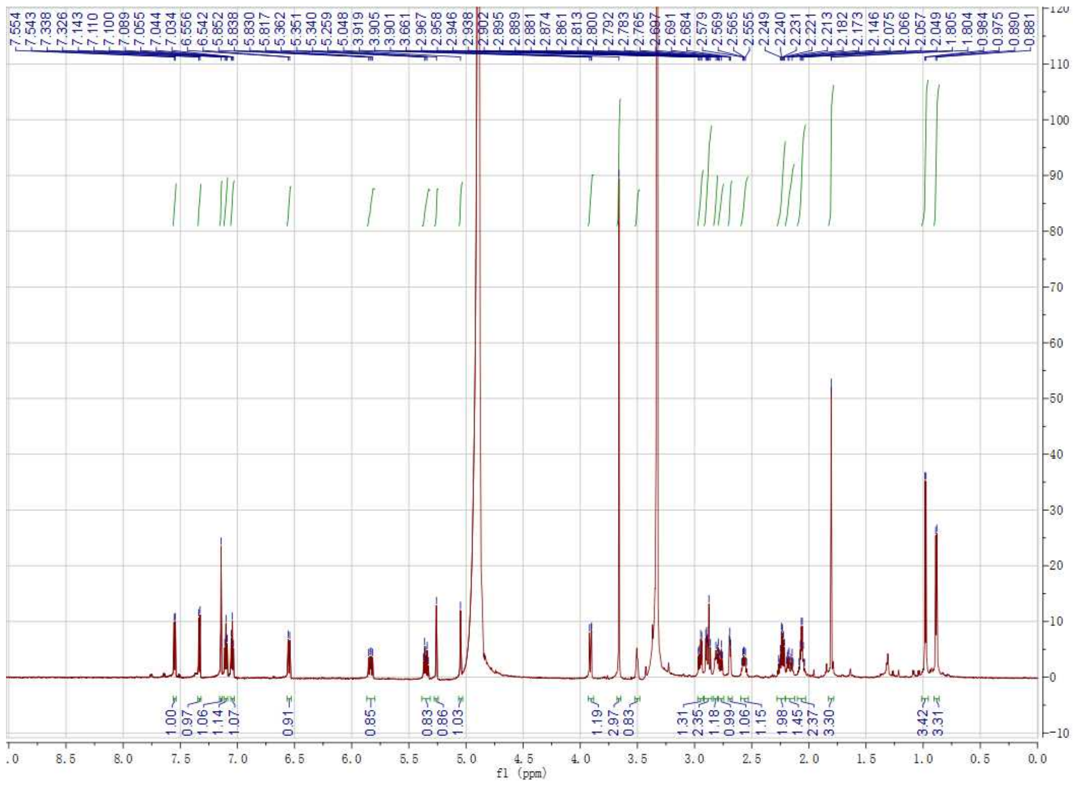

Figure S10. ^{1}H NMR of compound **2** (in CD_3OD)

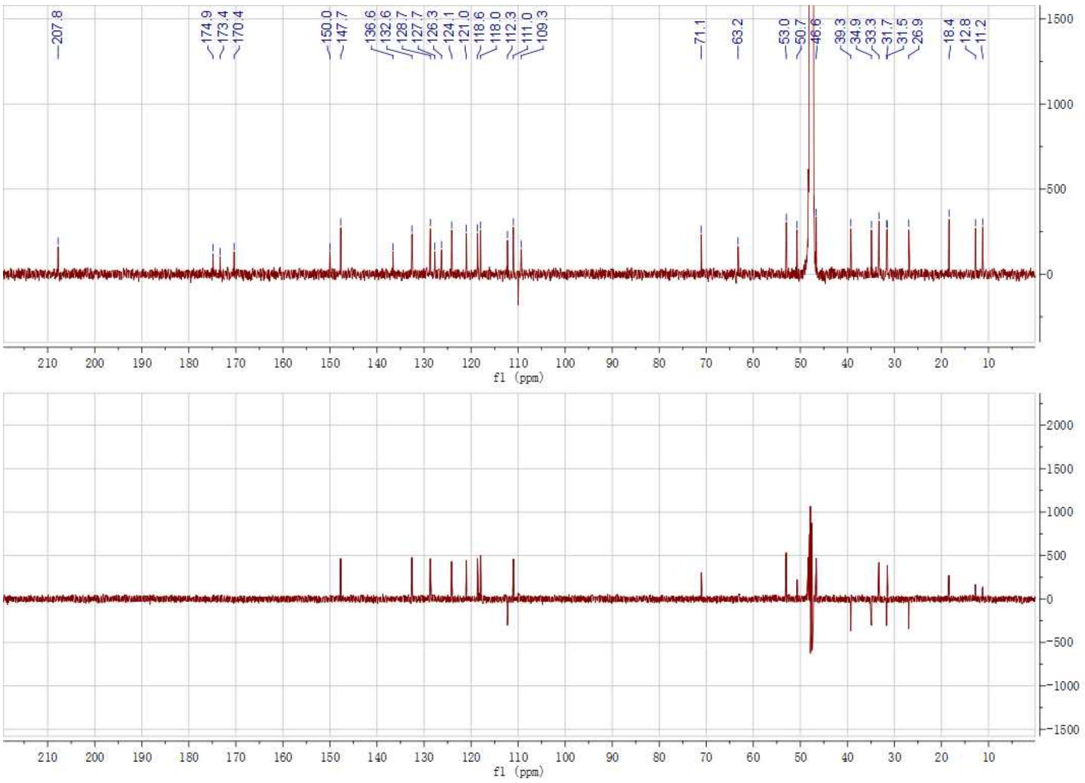

Figure S11. ^{13}C NMR of compound **2** (in CD_3OD)

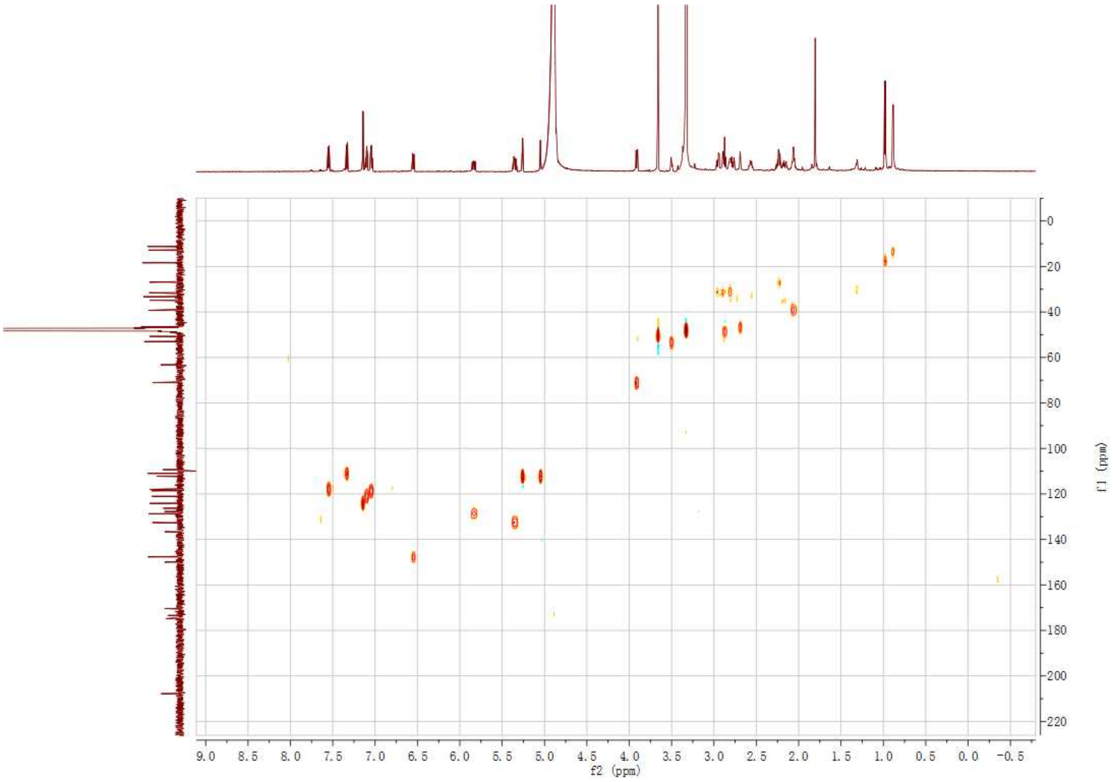

Figure S12. HSQC of compound **2** (in CD_3OD)

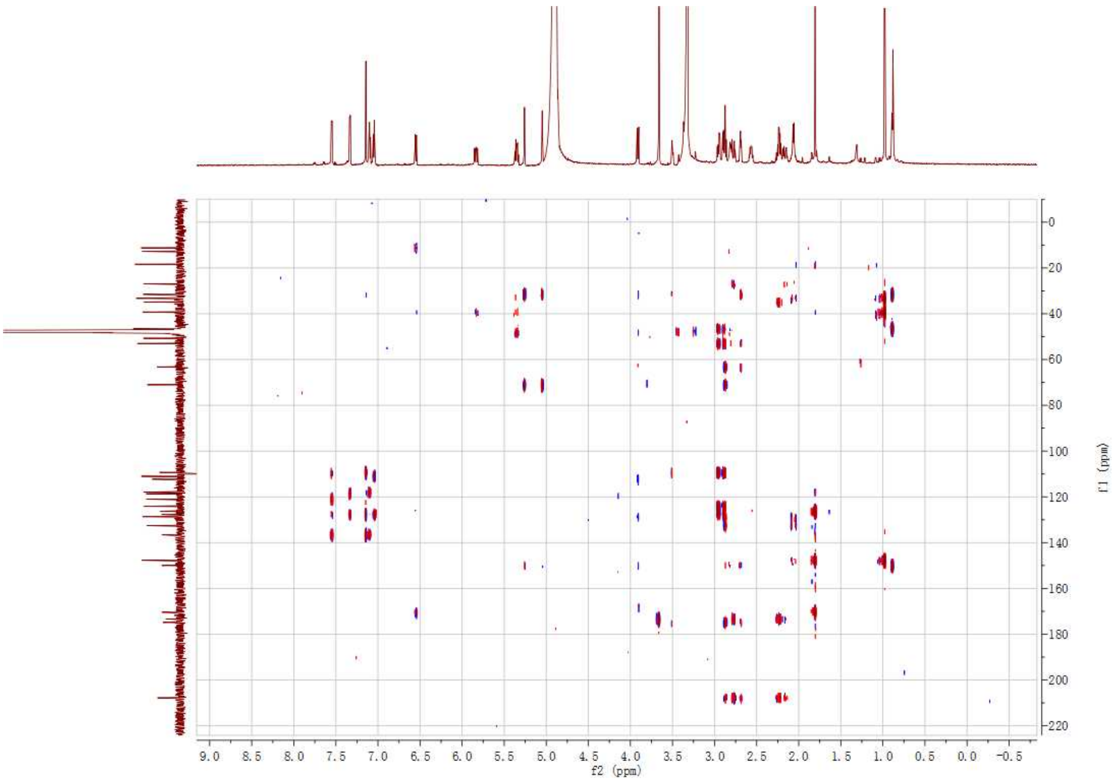

Figure S13. HMBC of compound **2** (in CD_3OD)

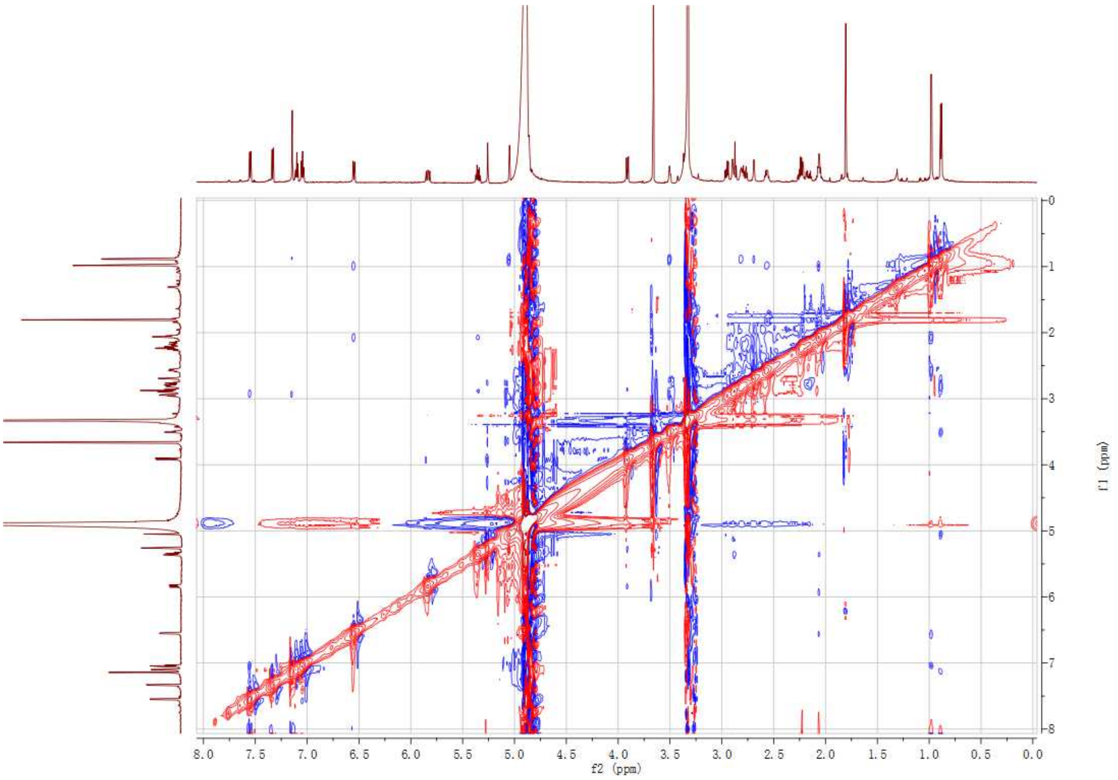

Figure S14. NOESY of compound **2** (in CD_3OD)

Mass Spectrum SmartFormula Report

Analysis Info

Analysis Name D:\Data\MS\data\202107\shaosurun_LTH1-15-3_pos_52_01_11334.d
Method LC_Direct Infusion_pos_70-500mz.m
Sample Name shaosurun_LTH1-15-3_pos
Comment

Acquisition Date 8/2/2021 3:04:33 PM
Operator SCSIO
Instrument maXis 255552.00029

Acquisition Parameter

Source Type	ESI	Ion Polarity	Positive	Set Nebulizer	0.4 Bar
Focus	Active	Set Capillary	4500 V	Set Dry Heater	180 °C
Scan Begin	70 m/z	Set End Plate Offset	-500 V	Set Dry Gas	4.0 l/min
Scan End	1500 m/z	Set Charging Voltage	0 V	Set Divert Valve	Waste
		Set Corona	0 nA	Set APCI Heater	0 °C

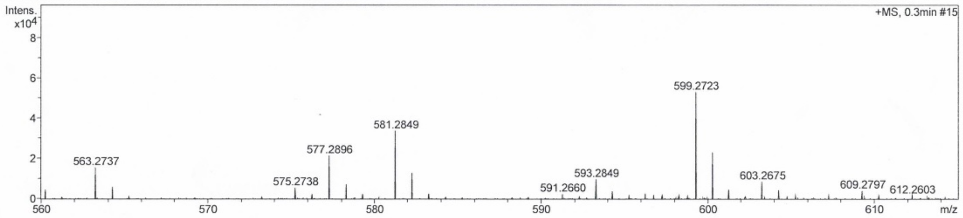

Meas. m/z	#	Ion Formula	Score	m/z	err [ppm]	err [mDa]	mSigma	rdb	e⁻ Conf	N-Rule
577.2896	1	C33H41N2O7	100.00	577.2908	-2.1	-1.2	24.0	14.5	even	ok
599.2723	1	C33H40N2NaO7	100.00	599.2728	0.8	0.5	32.4	14.5	even	ok
1153.5709	1	C66H81N4O14	100.00	1153.5744	3.0	3.5	558.7	28.5	even	ok
1175.5542	1	C66H80N4NaO14	100.00	1175.5563	1.8	2.2	217.1	28.5	even	ok

shaosurun_LTH1-15-3_pos_52_01_11334.d
Bruker Compass DataAnalysis 4.1 printed: 8/2/2021 3:14:45 PM by: SCSIO Page 1 of 1

Figure S15. HRESIMS of compound **2**

光谱峰值检测报告

2021/07/02 16:51:27

数据集: LTH1-15-3 - RawData

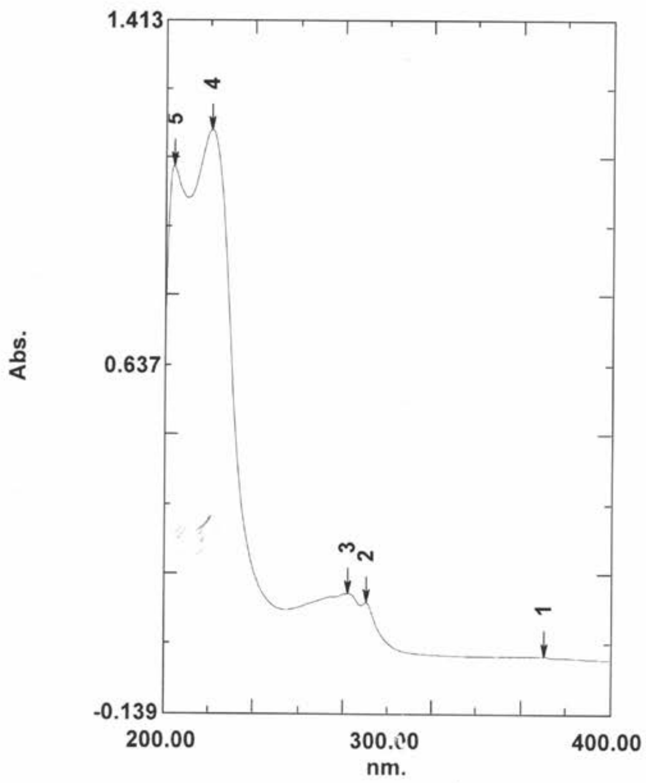

[测定属性]
波长范围(nm): 200.00 到 400.00
扫描速度: 中速
采样间隔: 0.2
自动采样间隔: 启用
扫描模式: 单个

[仪器属性]
仪器类型: UV-2600 系列
测定方式: 吸收值
狭缝宽: 2.0
积分时间: 0.1 秒.
光源转换波长: 323.0 nm
检测器单元: 直接
S/R 转换: 标准
阶梯校正: OFF

[附件属性]
附件: 无

[数据处理参数]
阈值: 0.0010000
点: 4
内插: 停用
平均: 停用

[样品准备属性]
重量:
体积:
稀释:
光程长:
附加信息:

No.	P/V	波长(nm)	Abs.	描述
1	⬆	370.80	-0.013	
2	⬆	290.20	0.106	
3	⬆	282.20	0.126	
4	⬆	220.60	1.164	
5	⬆	203.60	1.082	

Figure S16. UV of compound **2**

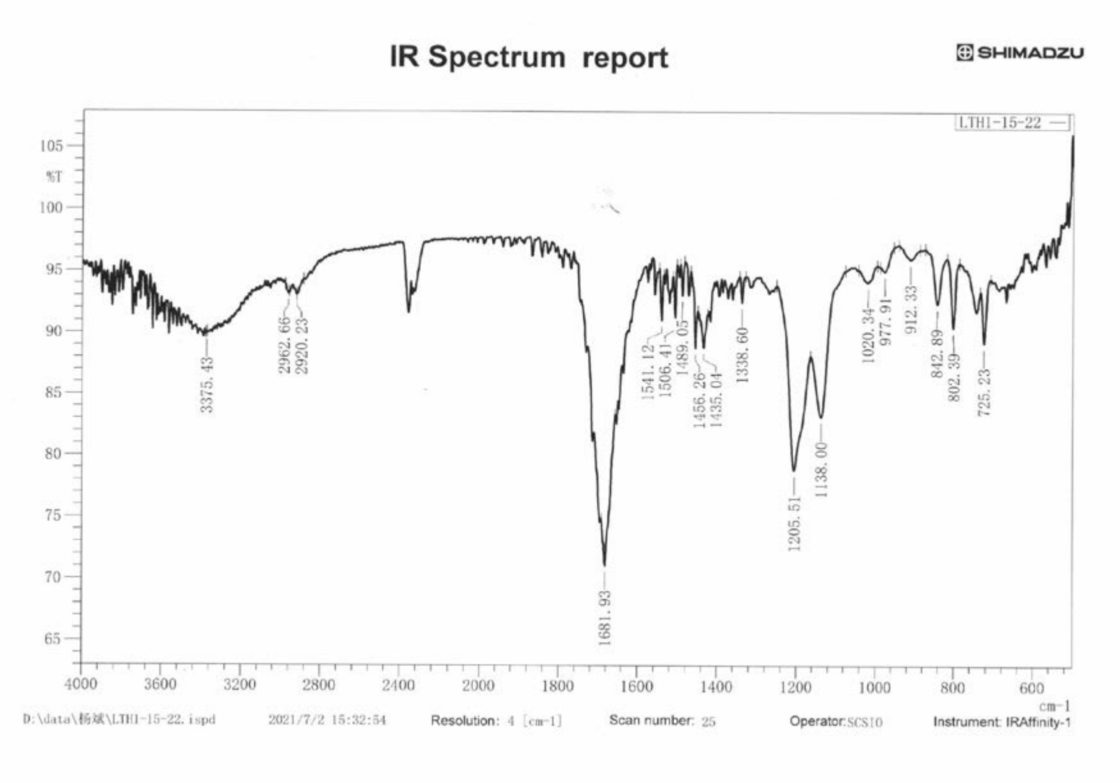

Figure S17. IR of compound **2**

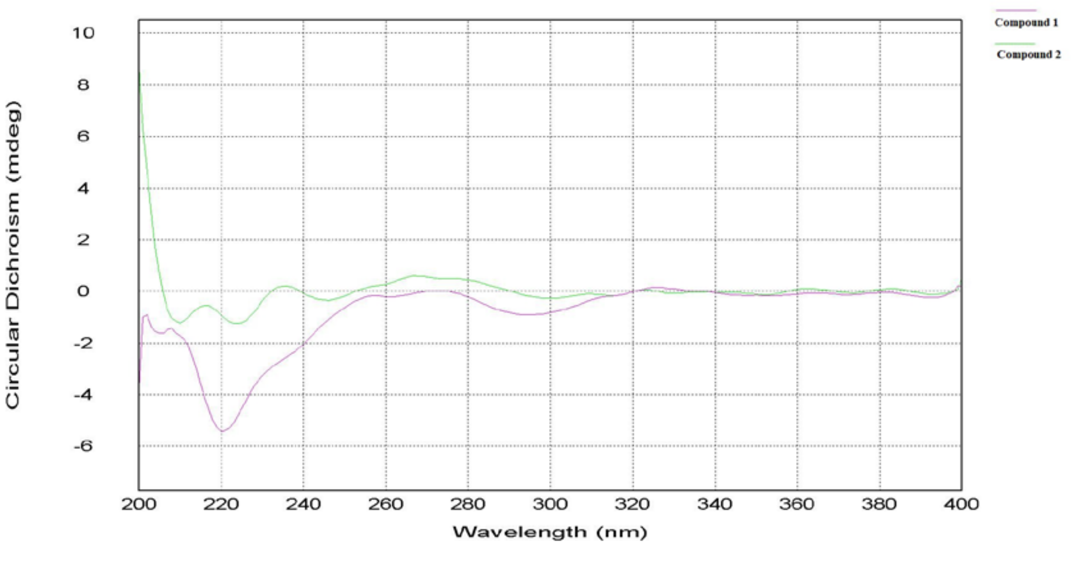

Figure S18. CD of compounds **1** and **2**

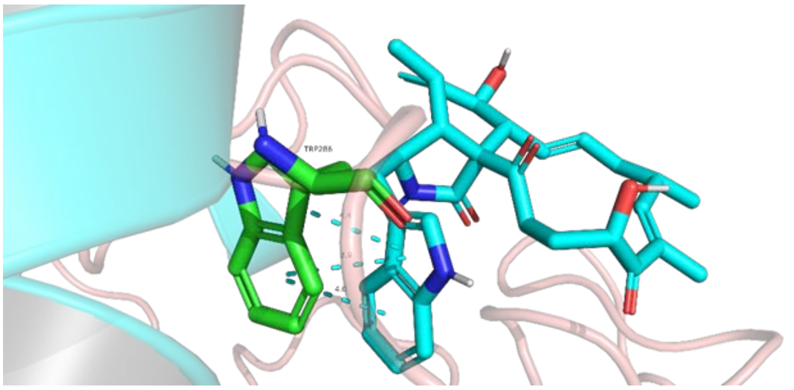

Figure S19. Molecular docking of chaetoglobosin Fex

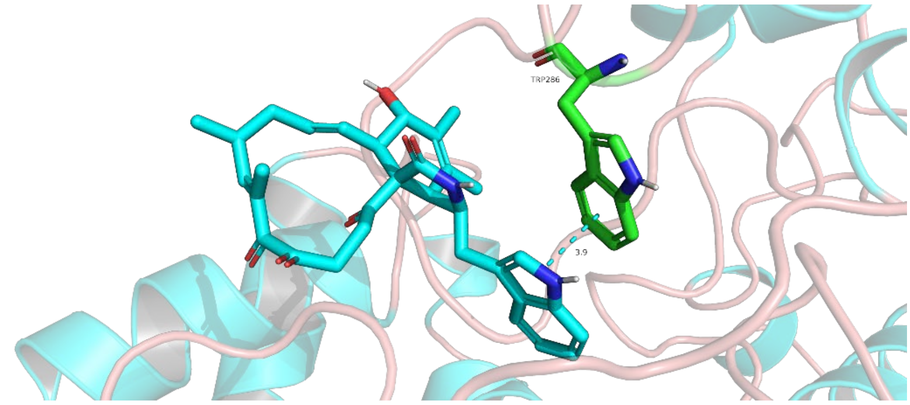

Figure S20. Molecular docking of chaetoglobosin G

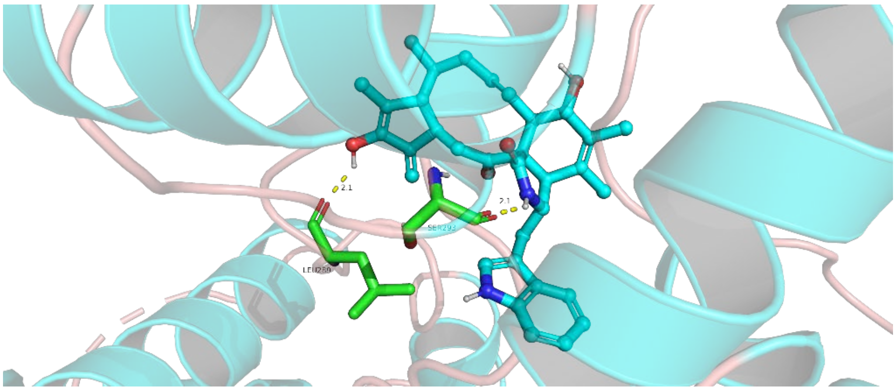

Figure S21. Molecular docking of chaetoglobosin Vb
